# Audio-visual combination of syllables involves time-sensitive dynamics following from fusion failure

**DOI:** 10.1038/s41598-020-75201-7

**Published:** 2020-10-22

**Authors:** Sophie Bouton, Jaime Delgado-Saa, Itsaso Olasagasti, Anne-Lise Giraud

**Affiliations:** 1 Department of Basic Neuroscience, University of Geneva, Biotech Campus, 9, Chemin des Mines, 1211 Geneva, Switzerland; 2 Centre de Recherche de l’Institut du Cerveau et de la Moelle Epinière and Centre de Neuro-Imagerie de Recherche, 75013 Paris, France; 3 Laboratoire Dynamique du Langage, CNRS and Université de Lyon UMR 5596, 69007 Lyon, France; 4 Biomedical Signal Processing and Artificial Inteligence Laboratory, Universidad del Norte, Barranquilla, Colombia

**Keywords:** Neuroscience, Cognitive neuroscience, Language

## Abstract

In face-to-face communication, audio-visual (AV) stimuli can be fused, combined or perceived as mismatching. While the left superior temporal sulcus (STS) is presumably the locus of AV integration, the process leading to *combination* is unknown. Based on previous modelling work, we hypothesize that *combination* results from a complex dynamic originating in a failure to integrate AV inputs, followed by a reconstruction of the most plausible AV sequence. In two different behavioural tasks and one MEG experiment, we observed that *combination* is more time demanding than *fusion*. Using time-/source-resolved human MEG analyses with linear and dynamic causal models, we show that both *fusion* and *combination* involve early detection of AV incongruence in the STS, whereas *combination* is further associated with enhanced activity of AV asynchrony-sensitive regions (auditory and inferior frontal cortices). Based on neural signal decoding, we finally show that only *combination* can be decoded from the IFG activity and that *combination* is decoded later than *fusion* in the STS. These results indicate that the AV speech integration outcome primarily depends on whether the STS converges or not onto an existing multimodal syllable representation, and that *combination* results from subsequent temporal processing, presumably the off-line re-ordering of incongruent AV stimuli.

## Introduction

Screen-based communication poses specific challenges to our brain for integrating audiovisual (AV) disparities due to either asynchronies between audio and visual signals (e.g. video call software) or to mismatching physical features (dubbed movies). To make sense of discrepant audio-visual speech stimuli, humans mostly focus on the auditory input^1^, which is taken as ground truth, and try to discard the disturbing visual one. In some specific cases, however, AV discrepancy goes unnoticed and the auditory and visual inputs are implicitly *fused* into a percept that corresponds to none of them^2^. More interestingly perhaps, discrepant AV stimuli can also be combined into a composite percept where simultaneous sensory inputs are perceived sequentially^2,3^. These two distinct outcomes can experimentally be obtained using the “McGurk effect”^2^, where an auditory /aba/ dubbed onto a facial display articulating /aga/ elicits the perception of a fused syllable /ada/, while an auditory /aga/ dubbed onto a visual /aba/ typically leads to a mix of the combined syllables /abga/ or /agba/. What determines whether AV stimuli are going to be fused^4–6^ or combined^7^, and the underlying neural dynamics of such a perceptual divergence is not known yet.

Audio-visual speech integration draws on a number of processing steps distributed over several cortical regions, including auditory and visual cortices, the left posterior temporal cortex, and higher-level language regions of the left prefrontal^8–12^ and anterior temporal cortices^13,14^. In this distributed network, the left superior temporal sulcus (STS) plays a central role in integrating visual and auditory inputs from the visual motion area (mediotemporal cortex, MT) and the auditory cortex (AC)^15–21^. The STS is characterized by relatively smooth temporal integration properties making it resilient to the natural asynchrony between auditory and visual speech inputs, i.e. the fact that orofacial speech movements often start before the sounds they produce^6,22,23^. Although the STS responds more strongly when auditory and visual speech are perfectly synchronous^24^, its activity remains largely insensitive to temporal discrepancies^25^, reflecting a broad temporal window of integration in the order of the syllable length (up to ~ 260 ms)^26^. This large integration window can even be pathologically stretched to about 1 s in subjects suffering from autism spectrum disorder^27^. Yet, the detection of shorter temporal AV asynchronies is possible and takes place in other brain regions, in particular in the dorsal premotor area and the inferior frontal gyrus^28–31^. The STS and the IFG regions hence exhibit different functions in AV speech integration^32,33^, owing to their different temporal integration properties^34^. Interestingly, a relative resilience to asynchrony could confer the STS a specific sensitivity to the incongruence of *physical* features across A and V modalities. A key function of the STS could hence be to resolve AV speech feature discrepancies^35^ via a process requiring a double sensitivity to canonical visual motion (lip movements) and auditory spectrotemporal (formant transitions) cues^32^. On the other hand, the frontal cortex has been widely associated with cognitive control^36^ and temporal information processing in both short- and long-term memory^37,38^. In the context of AV speech, the inferior frontal cortex is involved in monitoring AV temporal sequences^30^.

**Figure 1:**
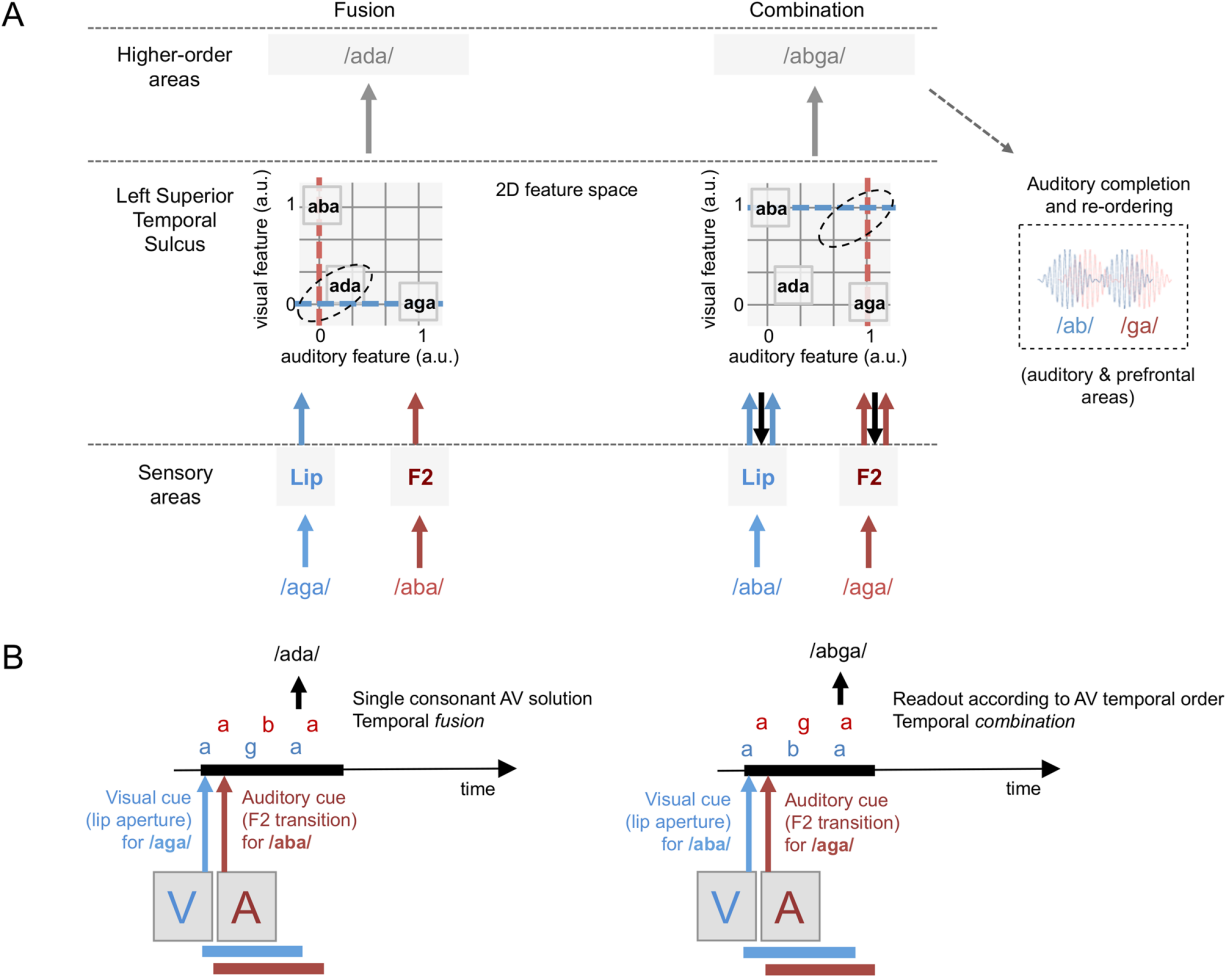
(**A**) Proposed neurophysiological mechanisms for *fusion* versus *combination*. We posit that after being processed by primary auditory and motion sensitive areas (bottom row), AV inputs converge in the left Superior Temporal Sulcus (STS, middle row) that works as a multidimensional feature space, here reduced to a simple 2D-space in which lip-motion and 2nd speech formant are the main dimensions. The STS is relatively insensitive to AV asynchrony [as depicted in (**B**)], but encodes both physical inputs in the 2D-space, converging on the most likely cause of a common speech source given these inputs. In the visual [aga]—auditory /aba/ condition, coordinates in the 2D space fall close to those of the existing syllable ‘ada’, which is picked as solution such that the subject senses no conflict. In the visual [aba]—auditory /aga/ condition, the absence of existing ‘aCa’ solution at coordinates crossing triggers post-hoc reconstruction of a plausible sequence of the inputs via complex consonant transitions (i.e., ‘abga’ or ‘agba’^1^). Both combination outputs require additional interaction with time sensitive (prefrontal and auditory) brain regions. Grey arrows represent the STS output as readout by higher order areas. Blue and red arrows represent visual and auditory inputs, respectively. (**B**) Discrepant audio (A) and visual (V) syllabic speech units ‘aCa’ are represented within a critical time-window for integrating them as a single item coming from the same source. The auditory percept is either a McGurk fusion ‘ada’ (left) or a combination percept (right). For combination, the percept is either ‘abga’ or ‘agba’ and it arises on-line from the real order with which each phoneme is detected. Image made using Microsoft PowerPoint, version 16.41.

To characterize the mechanism(s) underlying integration of A and V physical speech features (in the STS), we previously developed a generative predictive coding model^39^ that probed whether cross-modal predictions and prediction errors could be utilized to assemble speech stimuli into different perceptual solutions, corresponding to fused, i.e., /ada/, or combined, i.e., /abga/, percepts. The model showed that considering the temporal patterns in a 2nd acoustic formant/lip aperture two-dimensional (2D) feature space is sufficient to qualitatively reproduce participants’ behaviour for fused^18,40^ but also combined responses^39^. Simulations indicated that fusion is possible, and even expected, when the physical features of A and V stimulus, represented by the acoustic 2nd formant and lip aperture in the model, are located in the neighbourhood of an existing 2D syllable representation. This is the case for the canonical McGurk stimulus, which falls in the /ada/ neighbourhood, when the input corresponds to the /aga/ visual features and /aba/ auditory features. Conversely, audio-visual stimuli having no valid syllable representation in their 2nd formant/lip neighbourhood (Fig. 1A) lead to the sensation that the two (quasi) simultaneous consonants /b/ and /g/ are being pronounced sequentially, leading to the *combination* percepts /abga/ or /agba/^1,41,42^. Given the resilience to asynchrony of the STS, the *combination* process likely involves additional brain regions that are sensitive to AV timing.

These theoretical data lend support to the recent proposal that the neural processes underlying AV *combination* differ from those involved when AV stimuli are *congruent* and for AV *fusion*^7^. They also raise the issue of the neural process underlying AV *combination*, in particular how does the brain achieve sequential ordering of two phonemes occurring quasi simultaneously. In this study, we attempted to dissociate between two alternative hypotheses: (1) *combination* arises from a retrospect reconstruction of the most plausible AV sequence following from the impossibility for AV stimuli to converge on a single 2D (lip/formant) representation in the STS (Fig. 1A, right panel)^39,43^, or (2) *combination* readily involves fine detection of AV asynchrony and the percept arises on-line from the real AV phonemic order (Fig. 1B, right panel). To distinguish between these two scenarios, we interrogated the dynamics of human behavioural and MEG data. This required to not only present subjects with AV syllable stimuli leading to *fusion* and *combination*, but also to parameterize A and V asynchrony.

The delay to combine auditory and visual inputs should be similar for *fusion* and *combination* in the second hypothesis, but longer for *combination* than *fusion* in the first one, as time-sensitive processes leading an articulable phoneme sequence should follow from AV mismatch detection (Fig. 1A, right panel). Such a sequential process is expected to appear at the behavioural level, but most importantly in the timing of MEG neural signals. Further, if hypothesis 1 is correct, the pivotal role of the STS should translate into enhanced functional interactions with brain regions that are able to track AV timing during *combination* relative to *fusion*. Finally, for hypothesis 2 the ratio of ordered *combination* responses, e.g., /abga/ or /agba/, should vary according to the asynchrony direction, with more /abga/-type responses for audio-lag and more /agba/-type responses for audio-lead asynchronies, whereas for hypothesis 1 the /abga/ vs. /agba/ ratio should be independent from asynchrony.

## Results

To address whether combining AV stimuli results from on-line sensitivity to AV asynchrony or from *fusion* failure in the STS, we used the two canonical McGurk conditions, which give rise to either the fused percept ‘ada’ or ‘ata’, or a combined solution ‘abga’, ‘agba’, or ‘apka’, ‘akpa’. Although these stimuli are artificial (see Refs.^44–46^ for ecologically valid audiovisual stimulation), they allow for a rigorous parameterization of the various AV speech integration outcomes.

**Figure 2:**
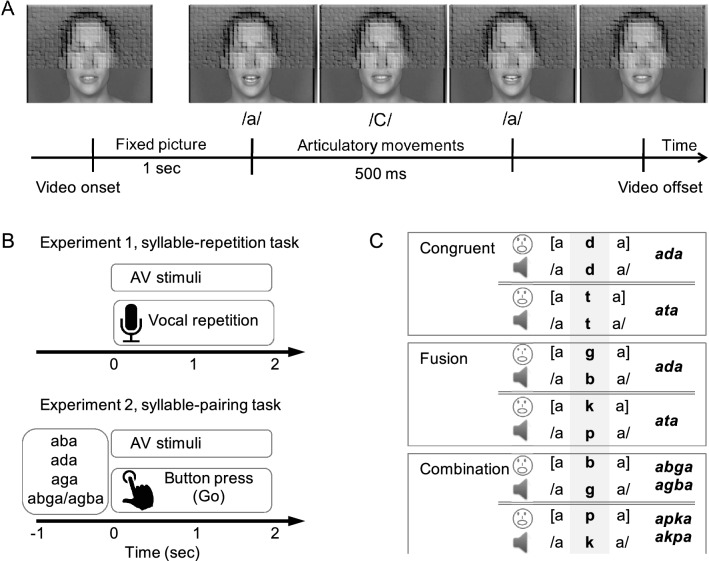
(**A**) Typical time course of audio-visual stimuli. (**B**) Example trials from experiments 1 and 2. Experiment 1: trials started with a 1 s fixation period, followed by a videoclip showing a speaker pronouncing a syllable. Participants had to repeat the perceived syllable as fast as possible. Experiment 2: trials started with a 1 s written syllable, followed by a short videoclip showing a speaker pronouncing a syllable. Participants were instructed to press a button as fast as possible if the written syllable matched the syllable they perceived from the AV videoclip. (**C**) Experimental conditions used in the behavioural and MEG experiments. The same three conditions, labelled ‘*congruent*’, ‘*fusion*’, and ‘*combination*’ were used in the behavioural and neuroimaging experiments. In each condition, stimuli combined a video track and an audio track, and used two consonant families: either ‘bdg’ or ‘ptk’. Right panel: the answer of interest (expected response depending on the exact AV stimulus integration) is shown in bold-italic. Image made using Microsoft PowerPoint, version 16.41.

We first ran two behavioural experiments carried out in distinct groups of participants. Both experiments involved vowel–consonant–vowel syllables of the type aCa denoted /aCa/ for audio and [aCa] for visual. These AV stimuli were used across three different conditions: (i) a *congruent* condition in which auditory and visual inputs corresponded to the same syllable (stimuli /ada/ + [ada] and stimuli /ata/ + [ata]), and two *incongruent* conditions in which auditory and visual inputs elicited either (ii) a *fusion* percept (stimuli /aba/ + [aga] and stimuli /apa/ + [aka]) or (iii) a *combination* percept (stimuli /aga/ + [aba] and stimuli /aka/ + [apa]) (Fig. 2C). All stimuli were video clips showing a female or a male speaker articulating aCa stimuli belonging to the ‘bdg’ or ‘ptk’ phoneme family. In a first experiment, 20 participants performed a repetition task. Instructions were the same as those given in the McGurk and MacDonald article (1976): participants watched the videos and were asked to report orally what they perceived as fast as possible (Fig. 2A,B), with no restrictions on the pronounced syllable. Detailed behavioural analyses are presented in the Methods section.

### AV *combination* takes longer than *fusion*

**Figure 3:**
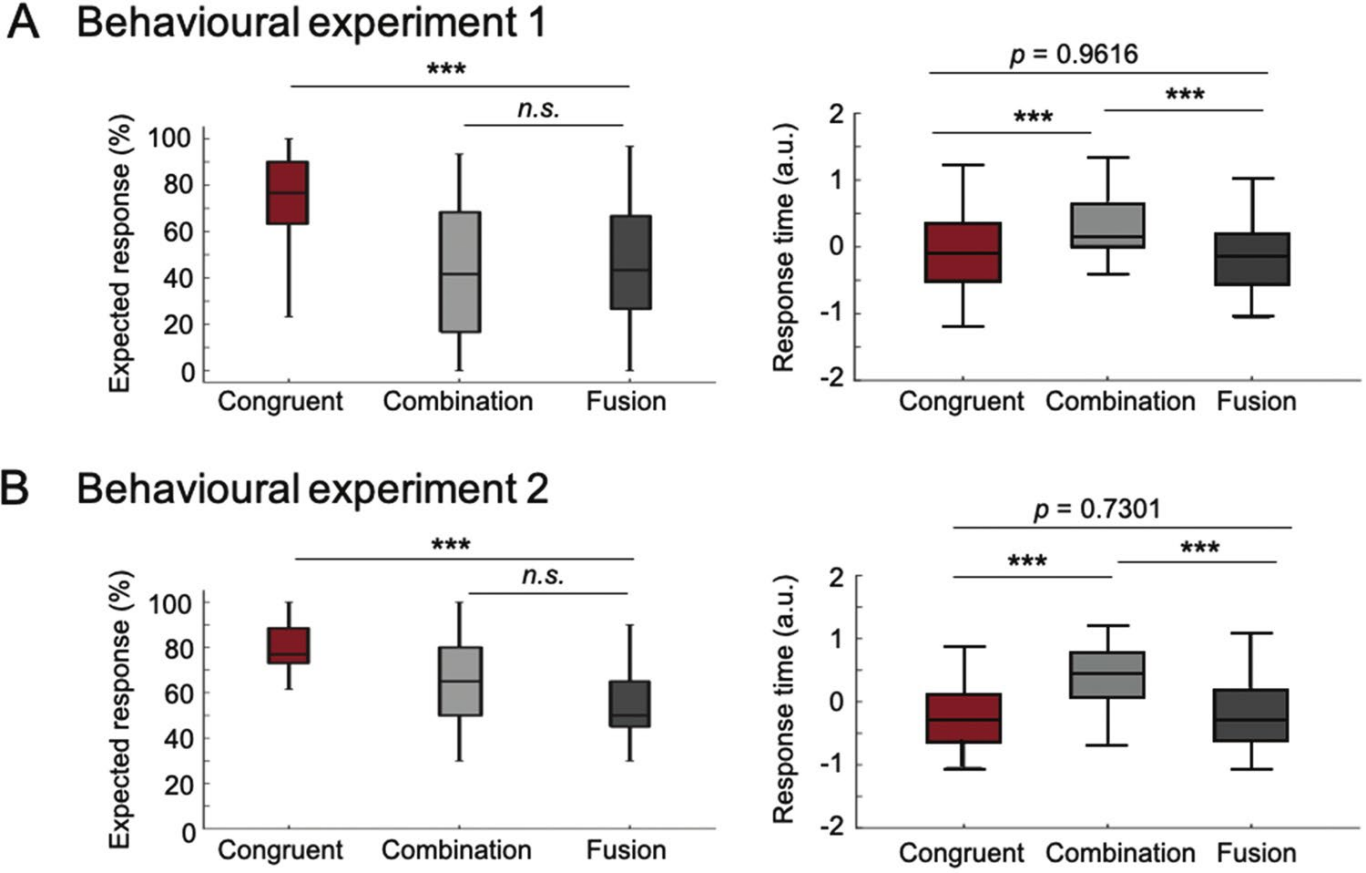
AV *combination* takes longer than *fusion*. Box Plots of (**A**) dependent variables from behavioural experiment 1, syllable-repetition task and of (**B**) dependent variables from behavioural experiment 2, syllable-pairing task. The box plots extend from the 25th to 75th percentiles, and whiskers extend to the full range of the data. The horizontal line in each box denotes the median. (**A**, **B**, left panels) Rate (%) of responses of interest in each condition, i.e., ‘ada’ or ‘ata’ responses in the *congruent* and *fusion* conditions, ‘abga’, ‘agba’, ‘apka’ or ‘akpa’ responses in the *combination* condition. (**A**, **B**, right panels) normalized and log transformed response time for responses of interest in each AV condition. In (**A**, **B**) error bars correspond to SD. Three stars indicate a significant difference at *P* < 0.001, two stars indicate a significant difference at *P* < 0.01, and n.s. indicate a non-significant difference (*P* > 0.05). Image made using Matlab 2019b.

We compared the task’s dependent variables (rate and delay of the responses of interest) between the three conditions (*congruent*, *fusion* and *combination*) using two repeated-measures ANOVAs (see “Methods”, Fig. 3, Supplementary Tables S1 and S2). In the repetition task (behavioural experiment 1), the rate for each response of interest (i.e. the percentage of ‘ada’ and ‘ata’ responses in the *fusion* and* congruent* conditions, and the percentage of ‘abga’, ‘apka’, ‘agba’ and ‘akpa’ responses in the *combination* condition) differed across conditions (*F*(2, 38) = 275.51, *P* < 0.001, partial η^2^ = 0.68), irrespective of the consonant family (*F* < 1) or speaker gender (*F* < 1) (Supplementary File 1B). Subjects reported more ‘ada’ and ‘ata’ responses in the *congruent* than *fusion* condition (*t*(19) = 8.45, *P* < 0.001, *power* = 98.84%) (Fig. 3A, left panel), but the mean rates for each response of interest were not different across *fusion* and *combination* conditions (*t*(19) = 0.69, *P* > 0.20, *power* = 28.26%). As expected, in the *fusion* condition participants mostly reported fused and auditory-driven responses. In the *combination* condition they reported mostly combined responses, but also auditory and visually-driven responses (Supplementary File 1B). In the three conditions, only response times (RTs) associated with the responses of interest were analysed, i.e., ‘ada’ and ‘ata’ responses in the *congruent* and *fusion* conditions, and ‘abga’–‘apka’–‘agba’–‘akpa’ responses in the *combination* condition. Log-transformed RTs differed between conditions (*F*(2, 239) = 7.99, *P* < 0.001, partial η^2^ = 0.12): the perceived syllable in the *fusion* condition was repeated as fast as in the *congruent* condition (*t* < 1, *power* = 4.98%), whereas the delay to pronounce the perceived syllables was longer in the *combination* condition than in the *congruent* and *fusion* conditions (*t*(39) = 4.61, *P* < 0.001, *power* = 53.37%, difference *combination* − *congruent* = 198 ms; *t*(39) = 4.16, *P* < 0.001, *power* = 52.36% difference *combination* − *fusion* = 191 ms, respectively) (Fig. 3A, right panel), irrespective of the consonant family (*F* < 1) or speaker gender (*F* < 1) (Supplementary File 1B). RTs thus indicate that subjects were slower to integrate mismatching audio-visual inputs when they elicited a combined rather than a fused, percept. Importantly, response time for ‘ada’ or ‘ata’ was equal whether it arose from congruent or incongruent syllables, showing that AV incongruence was not at the origin of the slower RT for *combination*.

Although, this first experiment supports the second hypothesis that participants should be faster to fuse than to combine AV stimuli, the effect could be biased by the difficulty to plan and articulate a more complex (double consonant) *combination* than a (single consonant) *fusion* syllable. To address this potential issue, we ran a second experiment in which 16 new participants performed a pairing task (behavioural experiment 2), where each trial included a written syllable followed by a video clip. Participants had to identify whether a syllable displayed on the screen before the video matched the syllable that was subsequently heard (Fig. 2B). Interestingly, the score for each response of interest in each condition was similar to that of the repetition task (Fig. 3B, left panel). Subjects were better at identifying a *congruent* than an *incongruent* stimulus (*F*(2, 30) = 30.26, *P* < 0.001, η^2^ = 0.27). In addition, participants matched the written syllable with the video faster in the *congruent* and *fusion* conditions than in the *combination* condition (*F*(2, 191) = 5.95, *P* < 0.001, η^2^ = 0.08; difference *combination* − *congruent* = 206 ms, *t*(31) = 4.47, *P* < 0.001, *power* = 31.92%; difference *combination* − *fusion* = 186 ms*, t*(31) = 7.83, *P* < 0.001, *power* = 26.05%; difference *fusion* − *congruent* = 20 ms, *t*(31) = 0.35 , *P* = 0.7301, *power* = 6.65%), confirming our previous findings (Fig. 3B, right panel), and showing that the extra delay for *combination* does not lie in added articulatory complexity. These data overall suggest that AV discrepancy was more easily solved in the *fusion* than in the *combination* condition, and that the integration of incongruent AV stimuli presumably relies on different neuronal processes depending on whether individuals end-up *fusing* or *combining* conflicting AV inputs.

### The phoneme order in *combination* is independent from AV asynchrony

**Figure 4:**
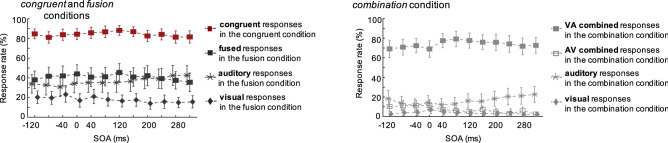
MEG experiment, behavioural results. Response rate depending on the stimulus onset asynchrony between visual and auditory stimuli. A negative stimulus onset asynchrony indicates that the auditory leads the visual input whereas a positive stimulus onset asynchrony shows that auditory lags visual. Error bars correspond to SEM. The left panel shows responses in *congruent* and *fusion* conditions: response of interest rates in *fusion* and *congruent* conditions (filled squares), auditory response rate in *fusion* condition (dark grey stars), and visual response rate in *fusion* condition (dark grey diamonds). The right panel shows responses in the *combination* condition only: response of interest rate, i.e., VA (visual-auditory) combined responses (light grey filled squares) and AV (audio-visual) combined responses (light grey squares) in *combination* condition, auditory response rate in *combination* condition (light grey stars), and visual response rate in *combination* condition (light grey diamonds). Image made using Matlab 2019b.

To investigate the neural underpinnings of AV *combination*, we recorded brain activity during perception of congruent and incongruent AV stimuli using magnetoencephalography (MEG). Sixteen participants watched videos showing a speaker pronouncing a syllable and reported which syllable they heard among 5 alternatives (i.e., ‘aba’, ‘ada’, ‘aga’, ‘abga’, ‘agba’ in the ‘bdg’ family, and ‘apa’, ‘ata’, ‘aka’, ‘apka’, ‘akpa’ in the ‘ptk’ family). Subject’s responses were purposely delayed to avoid temporal overlap between perceptual/decisional processes and motor effects due to button press. Response times hence do not constitute relevant data here, and we only consider the response rates (Fig. 4), which were calculated for each condition including congruent responses (i.e., /ada/ or /ata/) in the *congruent* condition, fused responses (i.e., ‘ada’ or ‘ata’) in the *fusion* condition, and combined responses (either VA, i.e., ‘abga’ or ‘apka’, or AV, i.e., ‘agba’ or ‘akpa’) in the *combination* condition. To address whether the components of the AV integration brain network were primarily sensitive to AV asynchrony or to AV physical features (formants and lip motion), and how these two variables contribute to *combination* versus *fusion*, we also varied the delay between audio and visual syllables. We used 12 different stimulus onset asynchronies over a temporal window ranging from − 120 ms audio lead to 320 ms audio lag (40 ms step), a window corresponding to a range in which *fusion* responses are expected to dominate over the auditory driven responses^6^; hence maximizing *fusion* reports (Fig. 4). This range of asynchrony was chosen not to disrupt the integration process, as within this temporal window A and V inputs are perceived as simultaneous^26,47^. In this task, the response of interest rate differed between conditions (*F*(2, 30) = 15.99, *P* < 0.001), whatever the consonant family (*F*(1, 15) = 1.98, *P* = 0.16), speaker gender (*F* < 1) or AV asynchrony (F < 1) (see Supplementary Table S3 for detailed statistics).

### Enhanced neural activity in the STS and IFG in *combination*

**Figure 5:**
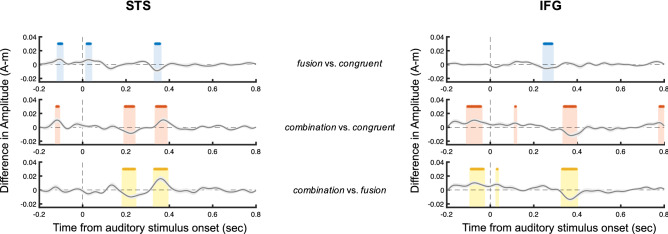
Differences in event-related activity between conditions, in two regions of interest, i.e. the STS and the IFG (*fusion* > *congruent* conditions in blue, *combination* > *congruent* conditions in red, *combination* > *fusion* conditions in yellow). The results for the other 4 ROIs are presented in Fig. S4. Stars indicate significant Student *t-test values that* were estimated in each difference: *fusion *vs.* congruent* conditions in blue, *combination *vs.* congruent* conditions in red, *combination *vs.* fusion* conditions in yellow (*P* < 0.05, corrected for multiple comparisons using FDR). Image made using Matlab 2019b.

After checking the global validity of the results in sensor space (Supplementary Fig. S1), we then used dynamic source modelling of MEG data to explore the global dynamics of AV *combination* relative to *fusion* and *congruent* conditions (Supplementary Fig. S2). We analysed the evoked activity in six regions of interest showing the strongest incongruence effect (incongruent > congruent): namely the left PAC (Primary Auditory Cortex), MT (Middle temporal visual area), STS (Superior Temporal Sulcus), STG (Superior Temporal Gyrus), IFG (Inferior Frontal Gyrus) and ATC (Anterior Temporal Cortex) (see Supplementary Fig. S3 for a spatial location of the corresponding scouts). Figure 5 shows basic contrasts of conditions for the STS and IFG (see Supplementary Fig. S4 for results in all regions). We found a statistical effect in both *combination vs. congruent* and *fusion vs. congruent* in the STS at ~ 100 ms pre-auditory stimulus onset (Fig. 5, blue and red), indicating that the STS could signal upcoming AV incongruence, presumably based on visual information (Refs.^35,48–51^, see also next data analyses). This was the only time point and location where *fusion* and *combination* showed a similar response profile (against congruent). High predictability of stimulus incongruence is possible in our experimental setting because the second input could be predicted from the first one: participants always received /ada/ + [ada] in the congruent condition, or /aba/ + [aga] in the *fusion* condition, or /aga/ + [aba] in the *combination* condition. Importantly, *combination* gave rise to a pre-audio activity in the IFG at − 80 ms different from both *congruent* and *fusion* conditions, followed by additional differences activity at + 200 ms in the STS and at + 350 ms in both the IFG and the STS. Figure 5 additionally shows a late effect in the IFG at ~ 750 ms post-auditory stimulus onset. Although all events cannot be explained by simple contrasts, these results provide a global picture of the event sequence at play in our experimental paradigm, and show differences in both STS and IFG at more points in time for *combination*.

### Enhanced connectivity from and to the IFG in *combination*

**Figure 6:**
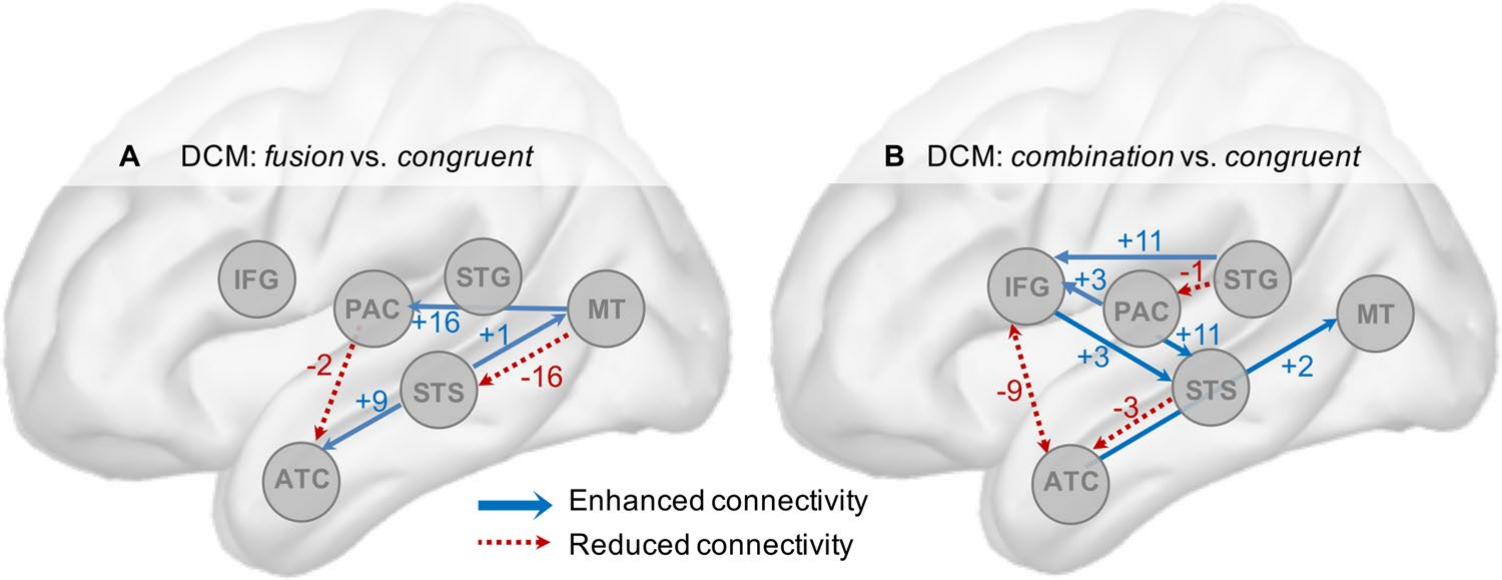
Dynamic causal modelling (DCM) of connectivity using event-related responses across the six main regions involved in audio-visual speech integration. The circles represent the sampled sources: primary auditory cortex (PAC), mediotemporal cortex (MT), the superior temporal gyrus (STG), the left superior temporal sulcus (STS), the inferior frontal gyrus (IFG) and the anterior temporal cortex (ATC). All connections and their values reflect enhanced or reduced connectivity of *fusion* (**A**) and *combination* (**B**) responses, relative to responses in the *congruent* condition. (**A**) *Fusion* was associated with increased connectivity from the STS to ATC and MT. Xonnectivity also increased from MT to PAC and decreased from PAC to ATC during *fusion.* (**B**) *Combination* was associated with increased connectivity from IFG and PAC to STS. Connectivity also increased from PAC to both STS and IFG, and decreased from STS to ATC, from ATC to IFG, from STG to PAC. We tested the differences between conditions using Parametric Empirical Bayes (PEB) models. Rather than comparing different network architectures, we performed a post-hoc search by pruning away parameters that did not contribute to the model evidence (*P* < 0.05). These results in a sparse graph where the connections shown are those that contributed significantly to the model evidence. Red dotted lines: reduced connectivity; Blue lines: enhanced connectivity. Image made using Microsoft PowerPoint, version 16.41.

Together, the behavioural results and source reconstructed MEG data indicate that *combination* required more computation than *fusion.* Overall the MEG results associated with fusion were closer to the congruent condition than those associated with *combination*. This suggests that combining incongruent AV stimuli might involve extra resources and perhaps a different neural network than fusing them. To further explore this hypothesis, we first probed directional functional coupling across the 6 previously defined regions of interest of the left hemisphere (ATC, IFG, STS, STG, MT, and PAC) using dynamic causal modelling (DCM). This analysis was not time-resolved, thus only showed directional connectivity patterns throughout the experimental trials. We found that *fusion* and *combination* had radically different neural dynamics, characterized by a dominant modulation of feed-forward and feedback connectivity from and to the STS, respectively. Importantly, we observed that connectivity from and to the IFG was only enhanced in *combination* (Fig. 6).

### Time-sensitivity and neural determinants of *combination* vs. *fusion*

The functional connectivity results confirm the central role of the STS in both *fusion* and *combination*, and the specific sensitivity of the IFG for *combination*. As hypothesized *combination* may be accounted for by two distinct neural mechanisms. The IFG could directly track the A and V inputs on-line, or it could reconstruct retrospectively the AV order after *fusion* failure in the STS. To address time-sensitivity during AV speech processing and enquire whether the *combination* outcome results from one or more specific neuronal event(s), we used a general linear model (GLM) on the 6 regions of interest. We searched for brain regions whose evoked activity was differently modulated by AV asynchrony and AV incongruence according to the syllable perceived (fused *vs.* combined output).

#### AV physical incongruence is first processed in the STS

**Figure 7:**
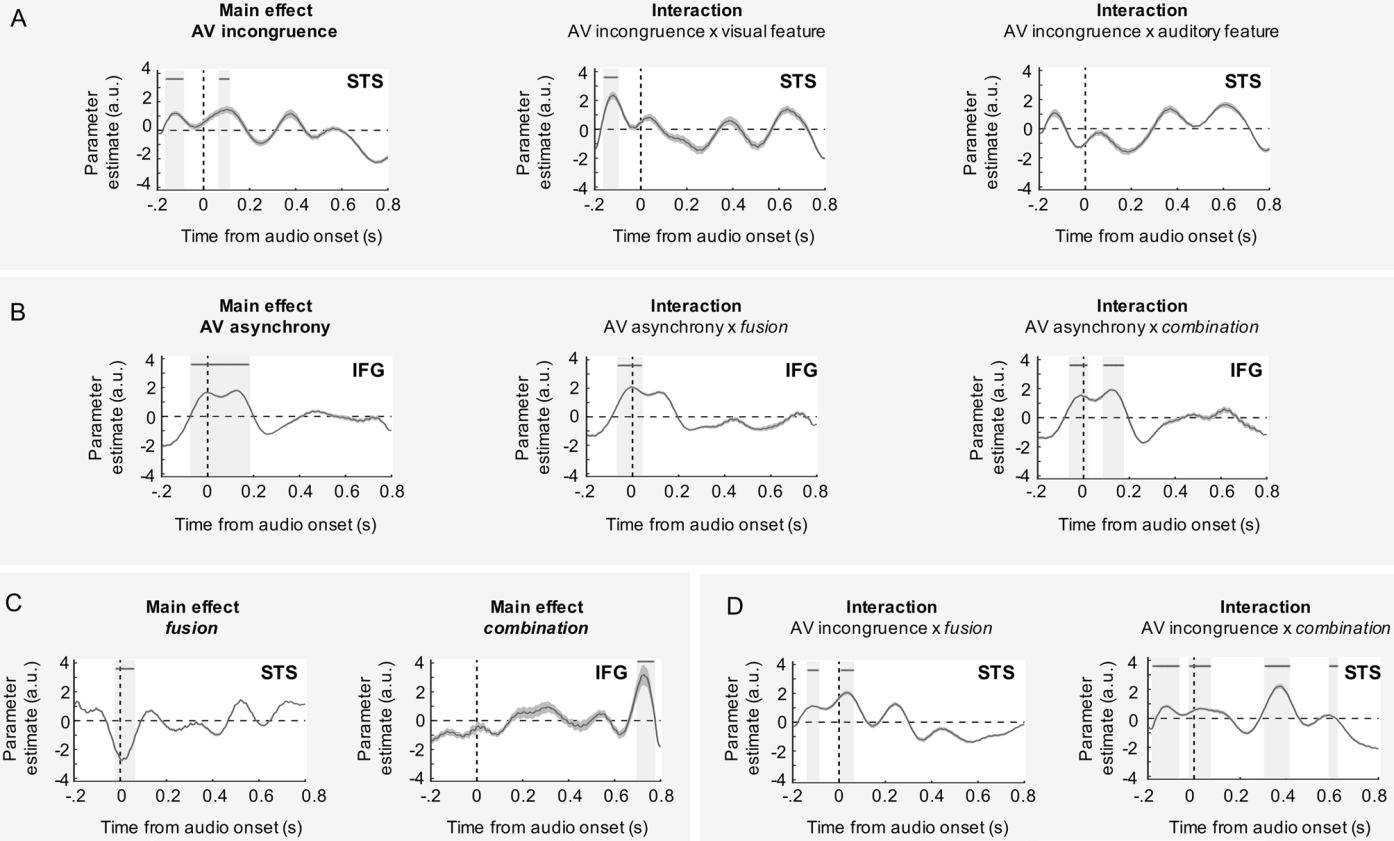
Results of the GLM analysis. The temporal dynamics of normalized beta in the STS and IFG. (**A**) AV Incongruence showed a significant effect in the STS before auditory onset, at the same time that the encoding of visual feature is significant. (**B**, **C**) The effect of AV asynchrony in the IFG was significant around − 50 ms until a fused percept is identified in the STS but last until 200 ms post-auditory stimulus onset when the AV inputs cannot be fused (i.e. in *combination* condition). (**C**, **D**) Combination was associated with a significant effect in the IFG at ~ 700 ms, after repeated incongruence X combination interaction effects at several time points in the STS. In contrast, *fusion* effects were significant only around the time of audio onset. Thick horizontal lines and light grey areas indicate time windows where parameter estimates diverge significantly from zero at a temporal cluster-wise corrected *P*-value of 0.05. The shaded error bounds indicate SD. *STS* superior temporal sulcus, *IFG* inferior frontal gyrus. Image made using Microsoft PowerPoint, version 16.41, and Matlab 2019b.

Consistent with the direct comparison of experimental conditions, the main effect of AV incongruence (Fig. 7A) was significant first in the STS during a time period ranging from 150 to 70 ms pre-audio onset. The high predictability of our stimuli presumably explains that cross-modal effects emerged very early in the STS, as previously shown^51,52^. Strong predictions likely resulted from overlearned AV associations and short-term adaptations likely to occur in the current experimental setting^53^. The effect of lip aperture was significant in the STS prior to auditory onset, presumably reflecting the prediction of subsequent incongruence (Fig. 7A and Supplementary Fig. S6). The interaction between lip aperture and AV incongruence was significant only before auditory onset whereas the interaction between auditory features and AV incongruence was never significant. Together, these results show that AV incongruence may reflect expectations before auditory onset but real AV integration process after auditory onset.

The effect of AV physical incongruence was significant in the STS ~ 50 ms after auditory onset (Fig. 7A), consistent with the contrast of evoked responses shown in Fig. 5. At this time, the second formant also had a significant effect in STS (Supplementary Fig. S6), suggesting that this effect could reflect a true integration process in the STS rather than mere expectation. Finally, while the incongruence effect in the STS was significant only at ~ 50 ms for *fusion*, it was also present at 400 and 600 ms in *combination* (interaction terms in Fig. 7D bottom and Supplementary Fig. S6).

#### AV asynchrony is first processed in the IFG

In line with recent literature, the effect of AV asynchrony was significant primarily in the IFG neural activity (20 ms pre- to 200 ms post-auditory stimulus onset), but also in PAC (around 300 ms post-auditory stimulus onset) (Fig. 7B and Supplementary Fig. S6). Crucially, in *fusion* the AV asynchrony effect in the IFG quickly dropped (50 ms post-audio), whereas in *combination* it occurred again at 100–200 ms (interaction terms in Fig. 7B). The recurrence of incongruence effects in the STS and asynchrony effects in the IFG in *combination* but not *fusion*, are in line with the behavioural findings indicating that *combination* is more time-demanding than *fusion*.

Finally, the specific *fusion* effects quickly dropped in the STS, likely reflecting convergence on an integrated solution, whereas in *combination* AV timing-sensitive activity reoccurred in the IFG along with an incongruence effect in the STS (Fig. 7C,D). PAC, IFG and STS exhibited recurrent activity until a complex (consonant sequence) solution could be elaborated (Supplementary Fig. S6). Crucially, the sequence of neural events in *combination* ended by a late effect in the IFG that did not reflect asynchrony tracking (Fig. 7B,C), but presumably a more abstract timing process, likely a retrospective ordering of the AV stimuli.

### Neural decoding of *combination* vs. *fusion*

Having established that the STS could quickly detect inconsistencies between auditory and visual physical features, and that the IFG was sensitive to AV asynchrony, we explored whether syllables could be decoded from neural activity. According to Hypothesis 1 (on-line AV integration) both combined and fused syllables should be decoded in the STS, whereas Hypothesis 2 (retrospect ordering after fusion failure in the STS) implies that combined syllables (i.e., ‘abga’, ‘agba’, ‘apka’ or ‘akpa’) might be decoded from neural activity in both the IFG and STS, and fused syllables (i.e., ‘ada’ or ‘ata’) only from the STS.

**Figure 8:**
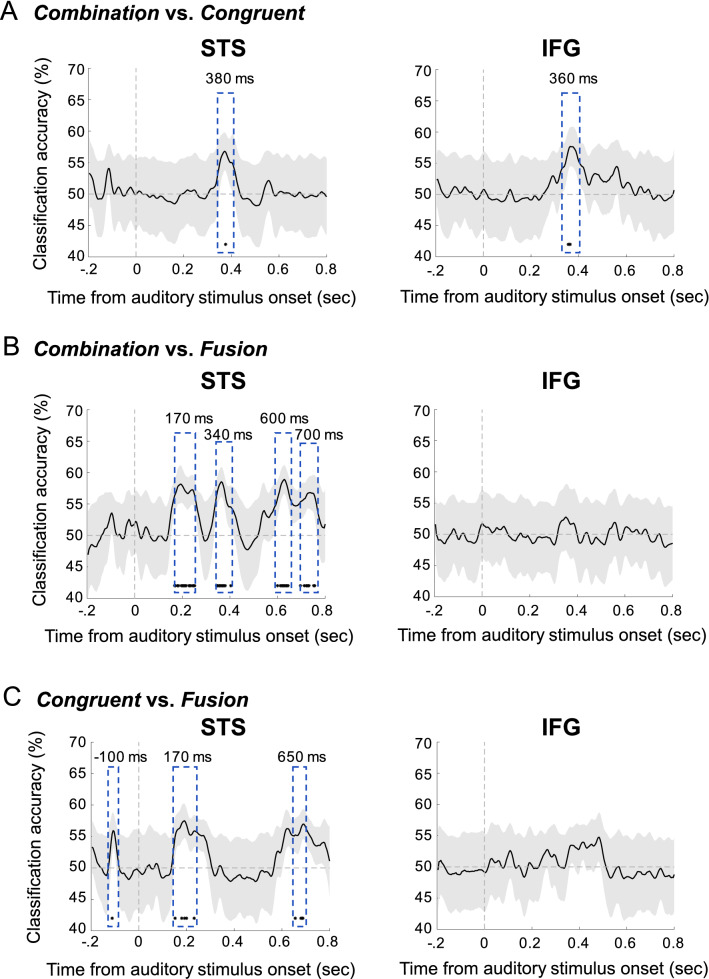
Decoding in the Left Superior Temporal Sulcus (STS) (left panels) and the Inferior Frontal Gyrus (IFG) (right panels). (**A**) Time course of univariate classification (accuracy) for ‘abga’, ‘agba’, ‘apka’ and ‘akpa’ responses from *combination* vs. ‘ada’ and ‘ata’ responses from *congruent*. Univariate classification between the responses of interest from *congruent* and *combination* conditions was possible in the IFG at ~ 360 ms and in the STS at ~ 380 ms. (**B**) Time course of univariate classification (accuracy) for ‘abga’, ‘agba’, ‘apka’ and ‘akpa’ responses from *combination* vs. ‘ada’ and ‘ata’ responses from *fusion*. Univariate classification of responses of interest from *fusion* and *combination* conditions was possible in the STS at ~ 170 ms, ~ 340 ms, ~ 600 ms and ~ 700 ms, but not in the IFG. (**C**) Time course of univariate classification (accuracy) for ‘ada and ‘ata’ responses from *congruent* vs. ‘ada’ and ‘ata’ responses from *fusion*. Univariate classification of responses of interest from *fusion* and *combination* conditions was possible in the STS at ~ − 100 ms, ~ 170 ms and ~ 650 ms, but not in the IFG. Image made using Microsoft PowerPoint, version 16.41, and Matlab 2019b.

We probed whether neural activity expressed in the STS and IFG held reliable information about AV stimulation conditions using three decoding analyses on the trials with responses of interest, to classify (1) ‘abga’, ‘agba’, ‘apka’ and ‘akpa’ responses from *combination* condition vs. ‘ada’ and ‘ata’ responses from congruent condition, (2) ‘abga’, ‘agba’, ‘apka’ and ‘akpa’ responses from *combination* condition vs. ‘ada’ and ‘ata’ responses from *fusion* condition, and (3) ‘ada’ and ‘ata’ responses from congruent condition vs. ‘ada’ and ‘ata’ responses from *fusion* condition (Fig. 8 and Supplementary Fig. S7). We used *time-resolved* decoding to keep track of the information propagation sequence. In line with previous findings^54^, we observed that local evoked activity from one region was sufficiently discriminable to permit syllable categorization using a maximum correlation coefficient classifier (see “Methods”). In the STS, three time-points appeared particularly interesting. First, at ~ 170 ms and ~ 650 ms post auditory stimulus onset both neural responses related to *combination vs. fusion* and neural responses related to congruent *vs. fusion* could be discriminated (Fig. 8B and BC). Second, at ~ 380 ms it was the neural responses related to *combination *vs. congruent and *combination vs*. *fusion* that could be separated (Fig. 8A,B). Importantly, neural responses related to *combination *vs.* congruent* could also be discriminated in the IFG at ~ 360 ms.

These analyses confirmed two crucial points: (1) *combination* was the only condition that could be decoded from neuronal activity in the IFG in addition to the STS, confirming the crucial implication of IFG when combining discrepant AV inputs; (2) *combination* is decodable after *fusion* in the STS since both fusion decoding analyses are significant at ~ 170 ms, while both *combination* decoding analyses are significant at ~ 380 ms. These results suggest that from ~ 170 ms the STS activity is susceptible to trigger a sequence of events leading to the identification of a combined syllable.

## Discussion

While the mechanisms leading to AV speech *fusion* are relatively well understood, those leading to AV stimulus *combination* are still unknown. Based on a previous computational model, we conjectured that *combination* follows from the difficulty to map the auditory and visual physical features in a multisensory space presumably located in the left STS^39^. *Combination* would hence result in a processing sequence that requires more computation than *fusion*, possibly involving retrospective temporal ordering of the auditory and visual input. The model was adapted to McGurk stimuli, and hence worked well with only lip and 2nd formant values (even though more features are likely at play in AV speech integration^55^). According to this simple 2-dimensional model, *fusion* occurs when the physical features of discordant AV stimuli fall in the vicinity of those corresponding to a lexically plausible and simple (single consonant transition) speech representation, whereas *combination* occurs when the physical features do not find AV matching features (Fig. 1A). In this view, after *fusion* failure, *combination* demands additional processing, possibly by frontal areas, to covertly generate a plausible articulatory sequence consistent with the AV input. The alternative scenario would be that combined percepts arise on-line from the sequential integration of the actual AV phonemic order since *combination* involves fine detection of AV asynchrony. The two scenarios lead to distinct predictions regarding the delay with which a *combination* percept arises. In the first case, combining discrepant AV stimuli should take significantly longer than fusing them, i.e. the time needed for *fusion* failure and active generation of alternative operations such as ordering of A and V stimuli. In the second case, the time to fuse and combine AV discrepant stimuli should be about equal, because *combination* mostly depends on the on-line perception of the AV order (Fig. 1B).

### AV *combination* takes longer than *fusion*

Our behavioural findings reveal longer delays for reporting AV *combination* than *congruent* and *fusion* percepts, a novel finding suggesting that reporting AV *combination* requires extra processing resources. Although congruent AV speech expected to produce faster and more accurate responses than incongruent AV speech^56,57^, we did not confirm that AV *fusion* was more time demanding than responding to congruent AV syllables^4,15,58–62^. The difference between our results and previous ones is explained by the fact that contrary to previous studies^15,58–62^, we only analysed trials where subjects effectively experienced *fusion* while discarding failed *fusion* trials (~ 45% of the trials).

More importantly, the results of both behavioural studies consistently show that *combination* was more time demanding than *fusion* (+ 200 ms). The second behavioural study clarified that the effect was not imputable to added articulatory demands. Importantly, the classification of *fusion* from source-resolved MEG responses in the STS, i.e. the key region for integrating audio and visual speech information^20,63–65^, was possible ~ 200 ms before *combination* could be decoded.

### The phoneme order in combination is independent from AV asynchrony

The combination of visual [aba] and auditory /aga/ can take two forms^1^: one (‘abga’, 72% of the responses) that respects the real AV temporal order, and another one (‘agba’, 8% of the responses) that does not, as previously observed^41^. A similar rate of production of the two forms across asynchronies strongly supports a post-hoc process resulting from recursive activity across the STS and IFG, rather than from on-line sequencing, which would lead to a greater rate of 'abga' percept when visual input precedes auditory input and 'agba' percept when auditory input precedes visual input.

### The role of the STS in the *fusion*/*combination* dynamic divergence

Although it is established that the STS integrates the information coming from A and V modalities, it is still not known whether it processes similarly or differently AV stimuli leading to *fusion* or *combination*. Using the GLM approach we found that the STS was the first region to signal physical incongruence between the two sensory modalities. The incongruence effect was even anticipated by the STS based on prediction from the visual stimuli^50^. A possible explanation for this anticipatory effect is that the STS quickly estimates whether to expect a precise auditory input (strong visual prediction) or rather a set of possible auditory inputs (weak visual prediction)^35^. When visual prediction is weak (e.g., visual /aga/), the STS could more easily fuse auditory and visual inputs, whereas when visual prediction is strong (e.g., /aba/), perceived incongruence is potentially stronger, in some cases resulting in a *combination* percept. In other words, the AV *fusion* vs. *combination* outcome could partly depend on the confidence associated with the expected input^44^.

However, according to our predictive model of AV syllable integration^39^, the most important factor determining *fusion* is whether the two stimuli meet close to an articulatory valid syllable representation within the 2nd acoustic formant/lip aperture 2D space. In the McGurk *fusion* case, visual /aga/ and auditory /aba/ fall in the vicinity of the 2D /ada/ representation, which quickly appears as a valid solution. This scenario is supported by the decoding analyses showing that neural activity in the STS signals *fusion* ~ 200 ms before *combination*.

In the *combination* case, since there is no 2D syllable representation at visual /aba/ and auditory /aga/ coordinates, the STS cannot readily converge on a viable articulatory solution. Activity in the STS linked to AV incongruence in *combination* remained sustained until 600 ms; during this time lapse, *combination* was associated with increased connectivity from IFG and PAC to STS. These findings suggest that *combination* requires a tight coordination between the temporal areas and the IFG^45,50^, presumably to organize the temporal serialization of AV inputs.

### The inferior frontal gyrus tracks AV asynchrony

To further understand the nature of the processing delay for AV *combination* relative to *fusion*, we explored how much the IFG was sensitive to AV asynchrony, and whether other brain regions could be involved in on-line vs. retrospect ordering of A and V stimuli leading to *combination*. Our MEG experimental design hence involved different stimulus onset asynchronies. Although the behavioural effect of AV asynchrony was weak (Fig. 4), as expected within the chosen asynchronous range^26,47^, we found both left PAC and IFG to be sensitive to this parameter, to the point that a classification algorithm from the IFG activity could decode *combination*. This finding presumably reflects the implication of the IFG in perceptual speech tasks requiring precise temporal parsing or sequencing of speech signals^34,63,66^.

The frontal cortex maintains, monitors and organizes temporal representations^28,64^, and among frontal areas, regions traditionally considered to be involved in speech processing, such as the lower frontal cortex and the upper temporal cortex, can be modulated by the temporal complexity of auditory and visual inputs^30^. These findings are consistent with our observation that the early IFG involvement in AV integration does not relate to feature identification, but is specific to AV timing. The IFG tracked AV asynchrony, at least within the range used in the experiment (from − 120 ms auditory lead to 320 ms auditory lag), without translating into a misalignment sensation. This confirmed that this range of AV asynchrony is registered while being perceptually well tolerated^6,26,47^.

Finally, we observed that the asynchrony effect in the IFG was followed by a similar effect in PAC, a finding that fits well with the previous observation that regularity in tone sequences modulates IFG activity, and that the estimated intervals propagate from the IFG to PAC^65,67^. Our study hence confirms that the IFG originates descending signals about estimated precision or sensory stimulus predictability^68^. Temporal order sensitivity in the left IFG might for instance allow listeners to predict word order from syntactic information^69,70^, a functional role that is compatible with the retrospect sequentialization of AV stimuli.

### Enhanced connectivity from and to the IFG in *combination*

The functional connectivity results confirmed a central role of the STS in both *fusion* and *combination*. They further show that in *combination*, the STS receives information from higher-order regions that are more sensitive to the precise timing of the AV events than the STS, whose function is rather to temporally integrate them despite AV asynchronies. By contrast, in *fusion* the STS dispatches information (presumably about the identified syllable) to other brain regions for recognition and sensory representation updating.

### On-line AV temporal tracking, versus post-hoc temporal ordering in *combination*

When auditory and visual signals are well aligned, integrating AV syllables is an easy and relatively low-level process, but when they are asynchronous or physically incongruent, integration involves a more complex dynamic. Our findings show that *combination* results both from the identification of the incongruence between the auditory and visual features in the STS, and from the detection of fine AV asynchronies in the IFG. The event sequence unraveled by the GLM indicates that *fusion* and *combination* arise from a visual pre-activation of the STS followed by the tracking of the AV asynchrony in the IFG, which is either interrupted by the input of a compatible audio (*fusion*), or sequentially continued by the input of an incompatible audio (*combination*). Our results hence support that *combination* is triggered by a failure of AV *fusion* in the STS, and reflects a posteriori reconstruction of the most plausible AV sequence.

## Conclusion

The present findings contribute to delineate the hitherto unknown mechanisms of AV *combination.* By showing that *combination* percepts arise downstream from *fusion* in the IFG, which we found sensitive to AV asynchrony, these results unravel the dynamics leading to the elaboration of novel endogenous constructs that best explain the cause of AV stimuli. On the contrary, we found no behavioural or neurophysiological arguments for the alternative scenario in which AV *combination* would result from on-line temporal ordering of A and V stimuli.

## Methods

### Subjects

Twenty healthy subjects participated in the first behavioural experiment (9 males—age range 20–28 years), 16 subjects in the second behavioural experiment (9 males—age range 21–31 years), and 15 took part in the MEG study (10 males—age range 21–24 years). All participants were right-handed, French-native speakers, and had no history of auditory or language disorders. Each behavioural experiment consisted of 1 h-long session performed in a quiet room while the MEG experiment consisted of 2 sessions lasting 2 h each. All participants were paid for their participation. Ethics permission was granted by the ethics committee of the University Hospital of Geneva in Switzerland for the behavioural experiments (CEREH 13-117), and by the Inserm ethics committee in France (biomedical protocol C07-28) for the MEG experiment. All participants provided written informed consent prior to the experiment. All experiments were performed in accordance with relevant guidelines and regulations of the approving local ethics committees.

### Stimuli

We recorded natural speech consisting of a man’s and a woman’s face articulating syllables. These two native French-speaker pronounced the syllables /apa/, /ata/, and /aka/ or the syllables /aba/, /ada/, and /aga/. The two syllable continua vary according to the place of articulation; furthermore, syllables are voiceless in one continuum, i.e., /apa/, /ata/ and /aka/, and voiced in the other continuum, i.e., /aba/, /ada/, and /aga/. To preserve the natural variability of speech, we used 10 exemplars of each syllable pronounced. Movies were recorded in a soundproof room into a 720 × 480-pixel movie with a digitization rate of 25 frames per s (1 frame = 40 ms). Stereo soundtracks were digitized at 44.1 kHz with 16-bits resolution.

We created 3 movie categories, which corresponded to the 3 stimulation conditions. *Congruent* videos corresponded to the initial recorded movie of the syllables /ada/ or /ata/. All videos had the same length and lasted 1000 ms. Using the soundtrack, we homogenized the duration of the stimuli: the vocal burst of the first /a/ and the consonantal burst were aligned across videos. The length of the second vocalic part was slightly variable across stimuli. Incongruent *fusion* pairs were created by dubbing an audio /apa/ or /aba/ onto a video [aka] or [aga], respectively. Audio and video were merged from the same speaker. The new soundtrack (/apa/ or /aba/), was systematically aligned to the initial soundtrack (/aka/ or /aga/) based on the vocalic burst of the first /a/ and on the consonantal burst. Incongruent *combination* pairs were created by dubbing an audio /aka/ or /aga/ onto respectively a video [apa] or [aba], using the same alignment procedure.

Auditory and Visual parameters of each condition are shown in Figs. 1 and 2.

### Tasks design

Auditory-visual stimuli were presented using Psychophysics-3 Toolbox and additional custom scripts written for Matlab (The Mathworks, Natick, Massachussetts, version 8.2.0.701). Sounds were presented binaurally at a sampling rate of 44,100 Hz and at an auditory level individually set before the task via earphones using an adaptive staircase procedure. For each participant, we determined prior the experiments their auditory perceptual threshold corresponding to 80% categorization accuracy. The estimated sound level was used to transmit the stimuli (mean 30 dB above hearing level) during the behavioral experiments (Experiment 1 and experiment 2) and MEG experiment.

#### Experiment 1, repetition task

Participants were individually tested and were instructed to watch each movie and repeat what they heard as fast as possible. We used the same instruction provided by McGurk and MacDonald (1976). Participants were asked to repeat as fast as they can what they heard. We did not limit the possible answers to a limited set of syllables. Nevertheless, note that for the three conditions (*congruent*, *fusion* and *combination*), different responses were expected according to our hypotheses (Fig. 2C).

In this experiment, participants were presented with four blocks, each one containing one speaker gender (female or male voice) and one continuum (b-d-g or p-t-k). Each block presented 90 AV stimuli corresponding to 30 *congruent* stimuli (A[d]V[d] or A[t]V[t]), 30 *fusion* stimuli (A[b]V[g] or A[p]V[k]), and 30 *combination* stimuli (A[g]V[b] or A[k]V[p]), for a total of 360 stimuli per subject. Trials were randomly presented in each block, and blocks were randomly presented across participants.

The responses of each participant were later transcribed and coded by two independent coders. They achieve a reliability of Cohen’s K = 0.94. When the two coders disagreed, a third coder listened to the response and decided which of the two proposed code was correct. The three coders were speech-language pathology students.

Each response was recorded. The recording started at the beginning of the video and lasted 3 s after the end of the video. Each soundtrack was analysed to detect the syllable onset using a home-made script that detected the beginning of the slope of the amplitude of the envelope of each sound. To ensure a good detection, the experimenter explicitly asked the participants to avoid making mouth noises during the experiment.

#### Experiment 2, pairing task

Participants were individually tested and were instructed to read the syllable written on the screen, then to watch the movie and to press the space bar as fast as possible when the written syllable matched what they heard. We used a go/no-go paradigm in which the participants were asked to respond when the written syllable matched the syllable heard. In the b-d-g sessions, participants could read ‘aba’, ‘ada’, ‘aga’, ‘abga’ or ‘agba’. In the p-t-k sessions, participants could read ‘apa’, ‘ata’, ‘aka’, ‘apka’ or ‘akpa’.

In this experiment, participants were presented with four blocks, each one containing one speaker gender (female or male voice) and one continuum (b-d-g or p-t-k). Each block included 150 AV stimuli corresponding to 50 *congruent* stimuli (A[d]V[d] or A[t]V[t]), 50 *fusion* stimuli (A[b]V[g] or A[p]V[k]), and 50 *combination* stimuli (A[g]V[b] or A[k]V[p]), for a total of 600 stimuli per subject. In each condition, 20% of the stimuli were presented after the written item ‘aba’, 20% of the stimuli were presented after the written item ‘aga’, 20% of the stimuli were presented after the written item ‘ada’, 20% of the stimuli were presented after the written item ‘abga’, and 20% of the stimuli were presented after the written item ‘agba’. Trials were randomly presented in each block, and blocks were randomly presented across participants.

In the two behavioural experiments, participants were sat 1 m from the monitor, and videos were displayed centered on a 17-inch Apple MacBookPro laptop on a black background. Sounds were presented through earphones (sennheiser CX 275).

#### MEG experiment

Each continuum (/bdg/ and /ptk/) was delivered to participants in two independent sessions of 360 trials each. Participants were asked to perform an identification task. Each trial comprised one video (randomly chosen among the 3 conditions), followed by a 1 s silent gap; then, a response screen with ‘aba’, ‘ada’, ‘aga’, ‘abga’ and ‘agba’ in the b-d-g sessions, and ‘apa’, ‘ata’, ‘aka’, ‘apka’ and ‘akpa’ in the p-t-k sessions, were displayed. Syllables were randomly displayed from right to left on the screen to prevent motor preparation and perseverative responses. During MEG recording, the appearance of the response screen was randomly jittered 100, 300 or 500 ms after the silent gap. Participants indicated their response by moving a cursor under the syllables and pressing a key to select the chosen syllable as quickly as possible. Subject’s responses were purposely delayed to avoid temporal overlap between perceptual processes and motor effects due to button press. Response times hence do not constitute relevant data. To limit eye movements, subjects were asked to blink only after giving their motor response. After the response, a jittered delay varying from 3 to 5 s led to the next trial.

In the MEG experiment, participants were presented with two sessions of four blocks, each session being similar with each block containing one speaker gender (female or male voice) and one continuum (b-d-g or p-t-k). Each block presented 180 AV stimuli corresponding to 60 *congruent* stimuli (A[d]V[d] or A[t]V[t]), 60 *fusion* stimuli (A[b]V[g] or A[p]V[k]), and 60 *combination* stimuli (A[g]V[b] or A[k]V[p]). Trials were randomly presented in each block, and blocks were randomly presented across participants. For each of the conditions (*congruent*, *combination* and *fusion*), 12 stimulus onset asynchronies were created, in steps of 40 ms over a temporal window ranging from − 120 ms audio lead to 320 ms audio lag. Audio-visual asynchronies were created by displacing the audio file in 40 ms increments (frame unit) with respect to the movie file. The negative stimulus onset asynchronies refer to auditory stimulus preceding the visual stimulus onset, and the positive asynchronies refer to visual stimulus onset preceding the auditory stimulus onset. In total, participants saw 720 videos (3 conditions × 20 videos per conditions × 12 AV asynchronies).

### MEG recording and preprocessing

Brain signals were recorded using Neuromag Elekta with a total of 306 channels composed of 204 axial gradiometers and 102 magnetometers. Recordings were first preprocessed using signal-space separation through Neuromag software MaxFilter. This allows removing signal coming from outside of the electrode sphere which allows removal of EOG (electrooculography) and ECG (electrocardiography) interference among other sources of noise. Originally, signals were sampled at a rate of 1000 Hz. A zero-phase forward–backward high-pass Butterworth filter of order 4 was used to remove frequencies below 0.05 Hz. Signals were then re-sampled at 250 Hz for further pre-processing stages. An IIR low-pass filter (Chebyshev Type I of order 8) with a cut frequency of 100 Hz was used to avoid aliasing. Before MEG recording, headshape was acquired for each participant using Polhemus. After the MEG session, an individual anatomical MRI was recorded (Tim-Trio, Siemens; 9 min anatomical T1-weighted MP-RAGE, 176 slices, field of view = 256, voxel size = 1 × 1 × 1 mm^3^). MEG data were preprocessed, analyzed and visualized using dataHandler software (https://cenir.icm-institute.org/en/service/service-3/), the Brainstorm toolbox^71^ and custom Matlab scripts.

### Analysis

#### Experiment 1, repetition task

We recorded the participant’s vocal response using a microphone. No feedback was provided after each response. The response time was measured as the interval between video onset and start of the syllable repetition from the audio recording on each trial. We also assessed the identification choice made by participants, i.e., the syllable repeated, on each trial.

#### Experiment 2, pairing task

The number of correct pairing between the written syllable and the video that led to a response of interest served as the measure of syllable identification. The response time was measured as the interval between video onset and the button press on each trial.

In the two behavioural experiments, percentage of responses of interest and response onset latency were calculated for each condition. Percentage of responses of interest ([ada-ata] for *congruent* and *fusion* conditions, or [abga-apka-agba-akpa] for the *combination* condition) was averaged separately across Consonant Family (‘bdg’ and ‘ptk’), Speaker Gender (male and female), and Conditions (*congruent*, *combination* and *fusion*) factors. Response onset latency was calculated, normalized, log transformed and averaged based on responses of interest across each condition. We reported the percentage of congruent responses in the *congruent* condition (i.e. /ada/ or /ata/ responses), the percentage of visual (i.e., /aga/ or /aka/), auditory (i.e., /aba/ or /apa/) and fused (i.e., /ada/ or /ata/) responses in the *fusion* condition, the percentage of visual (i.e., /aba/ or /apa/), auditory (i.e., /aga/ or /aka/), VA combined (i.e., /abga/ or /apka/) and AV combined (i.e., /agba/ or /akpa/) responses in the *combination* condition.

#### Behavioural analyses

Analysis of variance. Percentage of responses was analysed within each experiment (experiments 1 and 2) using a 3 × 2 repeated-measures ANOVAs with Conditions (*congruent*, *fusion*, *combination*) and responses of interest ([ada-ata] responses for the *congruent* and *fusion* conditions, and [abga-apka-agba-akpa] responses for the *combination* condition) as within-subjects factors. For the mean response latency, we measured the interval between video and vocal response onsets, for each type of response of interest. A 3 × 1 repeated measures ANOVA was performed on response times (RTs) with Conditions (*congruent*, *combination* and *fusion*) as a within-subjects factor. All ANOVAs modelled the variables Speaker Gender (female and male), and Consonant Family (‘bdg’ and ‘ptk’) as fixed-factors so as to generalize the results obtained to each speaker and each consonant family tested. To compute post-hoc power analyses, we used a post-hoc computation of the likelihood to observe a significant effect given the current samples^72^.

#### MEG processing

Using structural data, brain models for each subject were build using Brain Visa Software^73^. Individual brain models were mapped to the ICBM-112 brain model template for group-level analysis. Data analysis was performed with Brainstorm^71^, which is documented and freely available for download online under the GNU general public license.

The data considered (trials) started 0.2 s before the auditory event and until 0.8 s after the auditory input (i.e., the first vowel /a/ in /aCa/). We only analysed trials without eye artefacts or jumps in the signal. These artifacts were first removed/reduced using Neuromag software MaxFilter, followed by a manual selection of trials by visual inspection of the MEG signals. We did not conduct the analyses at sensor level as the contribution from different sources is mixed and our aim was to describe the brain network involved in AV speech perception. Nevertheless, we performed two sanity checks on the sensor space. We verified that both the response evoked by visual stimuli and the response evoked by auditory stimuli showed usual activations in the two-dimensional topographical MEG sensor plot^74^. Each trial was baseline corrected using an interval starting 500 ms after the start of the fixed video, and lasting 500 ms.

Using the data segmented in trials, we computed forward models using overlapping-sphere method, and source imaging using weighted minimum norm estimates (wMNEs) onto pre-processed data, all with using default Brainstorm parameters. A classical solution to the forward model is to use an L2 regularized minimum-norm inverse^75^. The L2 regularization parameter was fixed to 0.1 such that the covariance matrix of the data is stabilized by adding to it an identity matrix that is scaled to 10% of the largest eigenvalue. The wMNEs included an empirical estimate of the variance of the noise at each MEG sensor, which brings both magnetometers and gradiometers into the same basic range of units, allowing the source estimation to be proceed with a combined array of 306 sensors (204 planar gradiometers and 102 magnetometers). The sources were restricted to the cortex and the cortex was modelled using 15,000 vertices. One dipole per vertex was modelled, with the orientation constrained to be orthogonal to the cortical surface.

#### Regions of interest (ROI)

Six regions of interest in the left hemisphere were selected based on the significantly activated brain areas revealed in the incongruent > congruent contrast and the anatomical atlas of Destrieux et al.^76^. First, we selected all the regions that had the largest M100 (∼ 110 ms) evoked responses in the incongruent conditions relative to the congruent condition. ROI analyses were carried out by performing t-test (incongruent > congruent) across subjects in order to ensure that the selected regions were those where increased neural activity was found either in *fusion* or *combination*. All regions involved in the processing of AV incongruence will be highlighted. The centre of each ROI was defined by searching for the maximal t-value within the brain regions masked by the Destrieux atlas. We then used Brainstorm’s interactive user interface to extend the ROI over the anatomically surrounding source. The selected ROIs were the left PAC (Primary Auditory Cortex), MT (Middle temporal visual area), STS (Superior Temporal Sulcus), STG (Superior Temporal Gyrus), IFG (Inferior Frontal Gyrus) and ATC (Anterior Temporal Cortex) (see Supplementary Fig. S3 for a spatial location of the corresponding scouts).

#### Evoked responses

Signals from each region of interest were extracted and analysed. Evoked responses were computed by averaging MEG signals after source reconstruction across trials for each time sample around stimuli, for each subject and each condition (i.e., *fusion*, *combination* and *congruent*) (Supplementary Fig. S2). We then contrasted the conditions by subtracting their respective ERP response, which allowed testing 3 contrasts: *fusion* minus *congruent*, *combination* minus *congruent*, and *combination* minus *fusion*. Differences in the ERP response were detected across conditions by performing *t* tests against 0. FDR corrections for multiple comparisons were applied over the dimensions of interest (i.e., time samples, regions-of-interest and conditions), using the Benjamini–Hochberg procedure. For illustration purposes, obtained time courses for differences were smoothed using bandpass filtering (1–40 Hz) and then averaged across subjects.

#### Dynamic Causal Modelling (DCM) analysis procedure

Analysis of functional connectivity through Dynamic Causal Modelling was performed to determine significant changes in connectivity strength across conditions. We performed this analysis using the data from the six regions of interest defined above. We modelled forward and backward connections between nodes without pre-specified information, i.e. connections between every pair of nodes were probed. Cross-trial effects were estimated using the congruent condition as a reference, hence modelling all changes in connections that are necessary to explain *fusion* and *combination* conditions. The number of modes for data selection was set to 8 and 1 DCT (Discrete cosine transform) component was used per mode. Given that the signals are in the source space, we set the spatial model parameter to LFP in SPM. We tested whether effective connectivity was different depending on the conditions using the Parametrical Empirical Bayes (PEB) method through SPM. This second level analysis assumes that all subjects have the same basic architecture (same DCM) but that there could be variations in cross-regional connectivity strength. Rather than creating different competing models, we used a search over reduced PEB models that are obtained by switching off connections in order to determine which ones do not contribute to model evidence. The iterative procedure stops when discarding any parameter starts to decrease model evidence. This greedy search allows thousands of models to be compared quickly and efficiently. Finally, a Bayesian Model Average (BMA) was calculated over the 256 models from the final iteration of the search over the reduced PEB models as proposed in Zeidman et al.^77^. Connection strengths for which the posterior probability of being non-zero is lower than 0.95 were considered non significant and therefore removed from the final model.

#### General Linear Model procedure

We created a single GLM and applied it to each time point and each ROI separately. This analysis was performed on single-trial event-related activity, on the trials with responses of interest. The GLM included five categorical regressors (“syllable family”, “speaker gender”, “AV incongruence”, “fusion” and “combination”, and three parametric regressors (“visual feature”, “auditory feature”, “asynchrony”). These regressors were hierarchically orthogonalized in the following order: (1) the first regressor (referred as the “syllable family” regressor) modelled the two classes of syllables used in this experiment (ptk or bdg); (2) the “speaker gender” regressor refers to the gender of the speaker in the video; (3) the “visual feature” regressor was defined as the median amplitude of lip motion of the 40 stimuli in each condition with values [1, 0.5, 0.35] for [*Combination*, *Congruent*, *Fusion*] (Supplementary Fig. S5); (4) the “auditory feature” regressor was defined as the median value of second formant after consonantal release of the 40 stimuli in each condition with values [1, 0.7, 0.55] for [*Combination*, *Congruent*, *Fusion*] (Supplementary Fig. S5); (5) the “asynchrony” regressor was defined as the time interval separating the auditory and visual inputs without taking into account which one came first (1 = 0 ms; 2 = − 40 and 40 ms; 3 = − 80 and 80 ms; 4 = − 120 and 120 ms; 5 = 160 ms; 6 = 200 ms ; 7 = 240 ms; 8 = 280 ms ; 9 = 320 ms durations); (6) the “AV incongruence” regressor refers to whether inputs were congruent or incongruent; (7) the “combination” regressor denoted the combination stimuli perceived as combined percept, i.e., ‘abga’ or ‘agba’ for the stimuli /aga/ + [aba] or ‘apka’ or ‘akpa’ for the stimuli /aka/ + [apa]; (8) the “fusion” regressor was defined as the fusion stimuli perceived as fused percept, i.e., ‘ada’ for the stimuli /aba/ + [aga] or ‘ata’ for the stimuli /apa/ + [aka].

We regressed single-trial MEG signals against these 8 regressors at successive time points from – 200 to 800 ms following auditory stimulus onset. The two first regressors (“syllable family” and “gender of the speaker” regressors were included as fixed-factors so as to generalize the results obtained to each speaker and each consonant family tested. In addition, the GLM included 6 regressors to account for the main effect of visual and auditory features, AV incongruence, AV asynchrony, fusion and combination and 6 interaction regressors to account for the effect between AV incongruence and visual feature, AV incongruence and auditory feature, AV incongruence and fusion, AV incongruence and combination, asynchrony and fusion, asynchrony and combination, on brain activity. Finally, obtained time courses for each regressor were averaged across subjects.

We next determined the time window where regressors were significantly different from zero. FDR corrections for multiple comparisons were applied over the dimensions of interest (i.e., time samples, regions-of-interest and number of regressors), using the Benjamini–Hochberg step-up procedure. For the sake of illustration, obtained time courses for parametric modulators in the GLM were smoothed using bandpass filtering (1–40 Hz) and then averaged across subjects.

#### Classification of syllable identity

Decoding analyses were performed with the Neural Decoding Toolbox^78^, using a maximum correlation coefficient classifier on evoked responses in each region of interest. Analysis was constrained to trials with the responses of interest. Three different classifiers were built: one classifier was used to detect the neural activity capable of distinguishing between the fused response in *fusion* condition (i.e., ‘ada’ and ‘ata’ responses) from the combined response in *combination* condition (i.e., ‘abga’, ‘agba’, ‘akpa’ and ‘apka’ responses). Another classifier was used to detect where neural activity allowed the distinction between the combined response in *combination* condition (i.e., ‘abga’, ‘agba’, ‘akpa’ and ‘apka’ responses) and the correct response in *congruent* condition (i.e., ‘ada’ and ‘ata’ responses). A third classifier was used to detect where neural activity allowed the distinction between the fused response in *fusion* condition (i.e., ‘ada’ and ‘ata’ responses) and the correct response in *congruent* condition (i.e., ‘ada’ and ‘ata’ responses). Comparing the results of the three classifiers allowed us to assess profile similarity between the classifiers. By comparing the curves of the classifiers, we could determine the presence of information that specifically correspond to *combination* or that are shared by several conditions.

In the decoding procedure, each classifier was trained to associate MEG data with corresponding stimulus conditions (for each trial, the identity of the syllable perceived), irrespective of asynchrony. The amount of relevant information in the MEG signal was evaluated by testing the accuracy of the classifier on a separate set of test data. We performed the analyses at each time point, within 1-ms non-overlapping bins.

Decoding analyses were performed on each ROI with a cross-validation procedure where the classifier is trained on a subset of the data, and then the classifier’s performance is evaluated on the held-out test data. For each decoding run, data from the selected trials were divided into sets of 10 trials, and the data from each set of 10 trials were averaged together (see Ref.^79^ for a similar procedure). Each decoding run was performed at the group level, pooling all subjects together (see Ref.^80^ for a similar procedure). For example, in the first decoding procedure, a classifier was trained to associate MEG signals with the participants’ responses (the identified syllable, i.e., ‘ada’ vs. ‘abga’) in *fusion* and *combination* conditions.

For each decoding analysis, the classifier was trained on the participant’s response. It computed the correlation between MEG data and the syllable identified at each time point, and was trained on 80% of the data, while its performance was assessed on the withheld 20% of the test data. The splitting procedure between training and test data was performed 100 times to reduce the variance in the performance estimate. The classifier computed the correlation between test vectors (i.e., randomly selected mean values of 10 trials in the ROI at each time point) and a vector created from the mean of the training vectors. Each test point took the label of the class of the training data with which it maximally correlated. The reported final classification accuracy is reported as the percentage of correct trials classified in the test set averaged over all cross-validation splits. We then assessed the time window where decision values between the two categories were significantly different from zero (*t* test against zero). FDR corrections for multiple comparisons were applied over the dimensions of interest (i.e., time samples, regions-of-interest and number of classifiers), using the Benjamini–Hochberg step-up procedure.

## Supplementary information

Supplementary Information.

## Data Availability

The behavioural data are available here: https://figshare.com/s/63c773e39a239b72c473 and https://figshare.com/s/0ca3cc102593616cb3b2; The MEG data are available here: https://figshare.com/s/a4ba48347905b0c0790f.
