# Supplementary material for: Audio-visual combination of syllables involves time-sensitive dynamics following from fusion failure
Source: Sci Rep. 2020 Oct 22;10:18009. doi: 10.1038/s41598-020-75201-7 (PMC7583249; doi:10.1038/s41598-020-75201-7)
Supplement: Supplementary file 1 — Supplementary Information. [file 41598_2020_75201_MOESM1_ESM.docx]

**Audio-visual combination of syllables involves time-sensitive dynamics following from fusion failure**

Sophie Bouton^1,2,3^, Jaime Delgado-Saa^1,4^, Itsaso Olasagasti^1^, and Anne-Lise Giraud^1^

^1^*Department of Basic Neuroscience, University of Geneva, Biotech Campus, 9, Chemin des Mines, Geneva 1211, Switzerland*

^2^*Centre de Recherche de l′Institut du Cerveau et de la Moelle Epinière & Centre de Neuro-imagerie de Recherche, Paris, 75013, France*

^3^*Laboratoire Dynamique du Langage, CNRS & Université de Lyon UMR 5596, 69007 Lyon, France*

^4^*Biomedical Signal Processing and Artificial Inteligence Laboratory, Universidad del Norte, Barranquilla, Colombia*

**Figure S1.** Response profiles on the MEG sensor space. (A) response evoked by visual stimuli (grand average across all conditions), (a video following a black screen) from activity measured with gradiometers (top) and magnetometers (bottom). (B) response evoked by auditory stimuli (grand average across all conditions), i.e. at the consonant burst of the stimuli, from activity measured with gradiometers (top) and magnetometers (bottom). Image made using Microsoft PowerPoint, version 16.41, and Matlab 2019b.


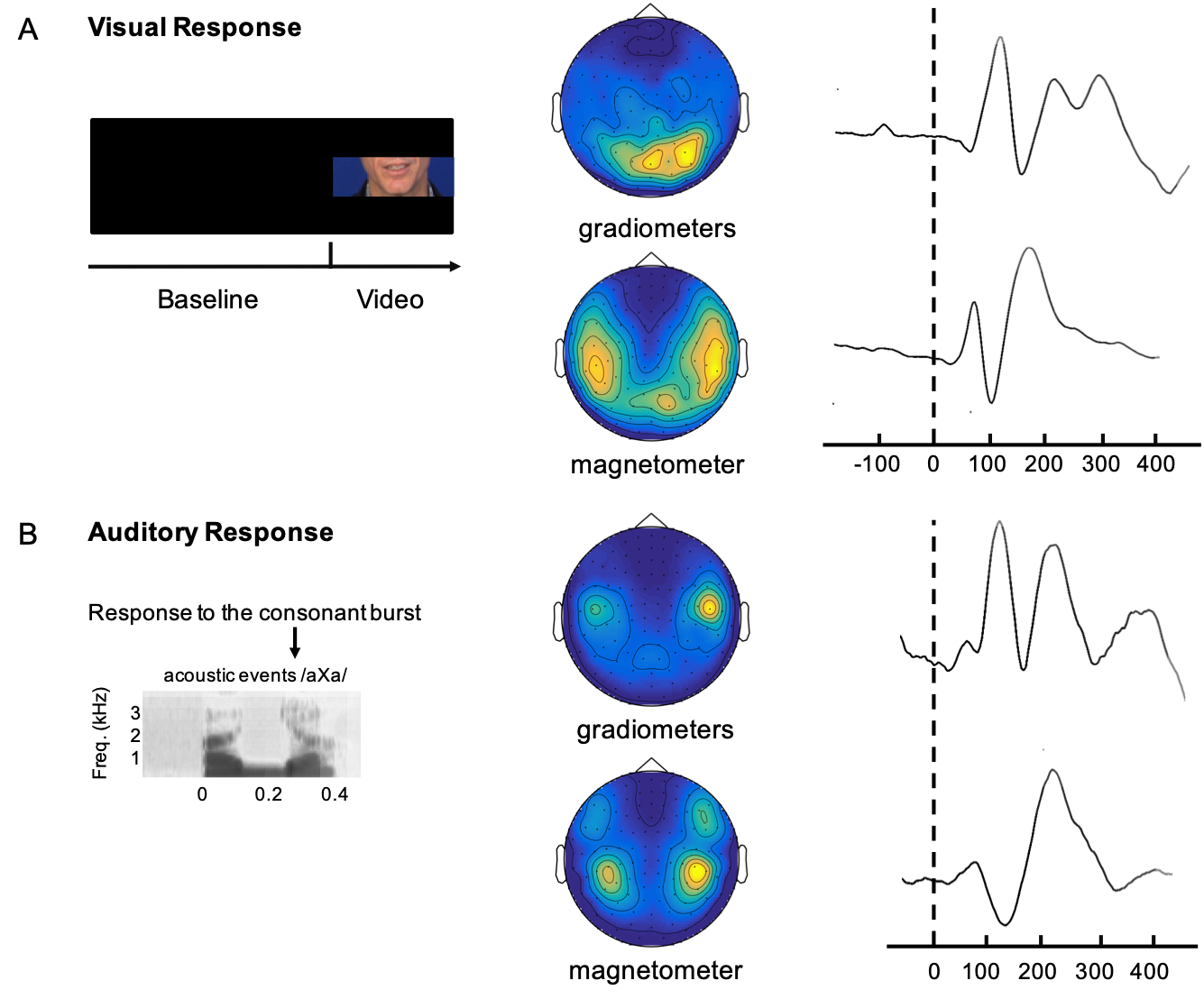


**Figure S2.** Evoked related field in each condition (congruent in blue, fusion in red, and combination in yellow) in each area of interest. The spectrograms are examples, used to illustrate how neuronal activity is locked to the auditory signal. Image made using Microsoft PowerPoint, version 16.41, and Matlab 2019b.

**Figure S3.** Regions of Interest are the left primary auditory cortex (PAC), the left mediotemporal cortex (MT), the left superior temporal gyrus (STG), the left superior temporal sulcus (STS), the left inferior frontal gyrus (IFG) and the left anterior temporal cortex (ATC). Image made using the Brainstorm toolbox^71^.

**Figure S4.** Differences in event-related activity between conditions, in six regions of interest, i.e. PAC, STG, STS, ATC, MT and IFG (fusion > congruent conditions in blue, combination > congruent conditions in red, combination > fusion conditions in yellow). Stars indicate significant Student t-test values that were estimated in each difference: fusion vs. congruent conditions in blue, combination vs. congruent conditions in red, combination vs. fusion conditions in yellow (P < 0.05, corrected for multiple comparisons using FDR). PAC. primary auditory cortex; STG. superior temporal gyrus; STS. superior temporal sulcus; ATC. Anterior Temporal Cortex; MT. MedioTemporal Cortex; IFG. inferior frontal gyrus. Image made using Matlab 2019b.

**Figure S5.** Stimulus features. Top panel: ‘bdg’ family. Right panel: measure of lip motion amplitude and 2^nd^ formant after consonantal release for each of the 20 stimuli in each stimulus category of the ‘bdg’ family. Diamonds show the median values for each stimulus type. The lip motion amplitude corresponds to the lip aperture difference between maximal aperture at the vowels and the minimal aperture upon consonant occlusion in the middle: ((max_lip aperture 1- min lip aperture) + (max lip aperture 2- min lip aperture)) / 2. The location of congruent aga and aba stimuli are given for reference, they were not presented to the participants in the study. Right panel: spectrogram for sample productions of /aba/, /ada/ and /aga/ sounds for the female speaker (left) and the male speaker (right). Bottom panel: same figures for the ‘ptk’ family. Image made using Microsoft PowerPoint, version 16.41, and Matlab 2019b.

**Figure S6.** Results of the GLM analyses in four area of interest (STS, IFG, STG and PAC). Thick horizontal lines and light grey areas indicate time windows where parameter estimates diverge significantly from zero at a temporal cluster-wise corrected p-value of 0.05. The shaded error bounds indicate s.t.d. STS. superior temporal sulcus; IFG. inferior frontal gyrus; STG. superior temporal gyrus; PAC. primary auditory cortex. Image made using Microsoft PowerPoint, version 16.41, and Matlab 2019b.

**Figure S7.** Decoding in the left primary auditory cortex (PAC), the left superior temporal gyrus (STG), the left superior temporal sulcus (STS), the left inferior frontal gyrus (IFG). (A) The time course of the (normalized) univariate classifier results for combination versus congruent. (B). The time course of the (normalized) univariate classifier results for fusion versus combination. (C). The time course of the (normalized) univariate classifier results for fusion versus congruent. Image made using Matlab 2019b.


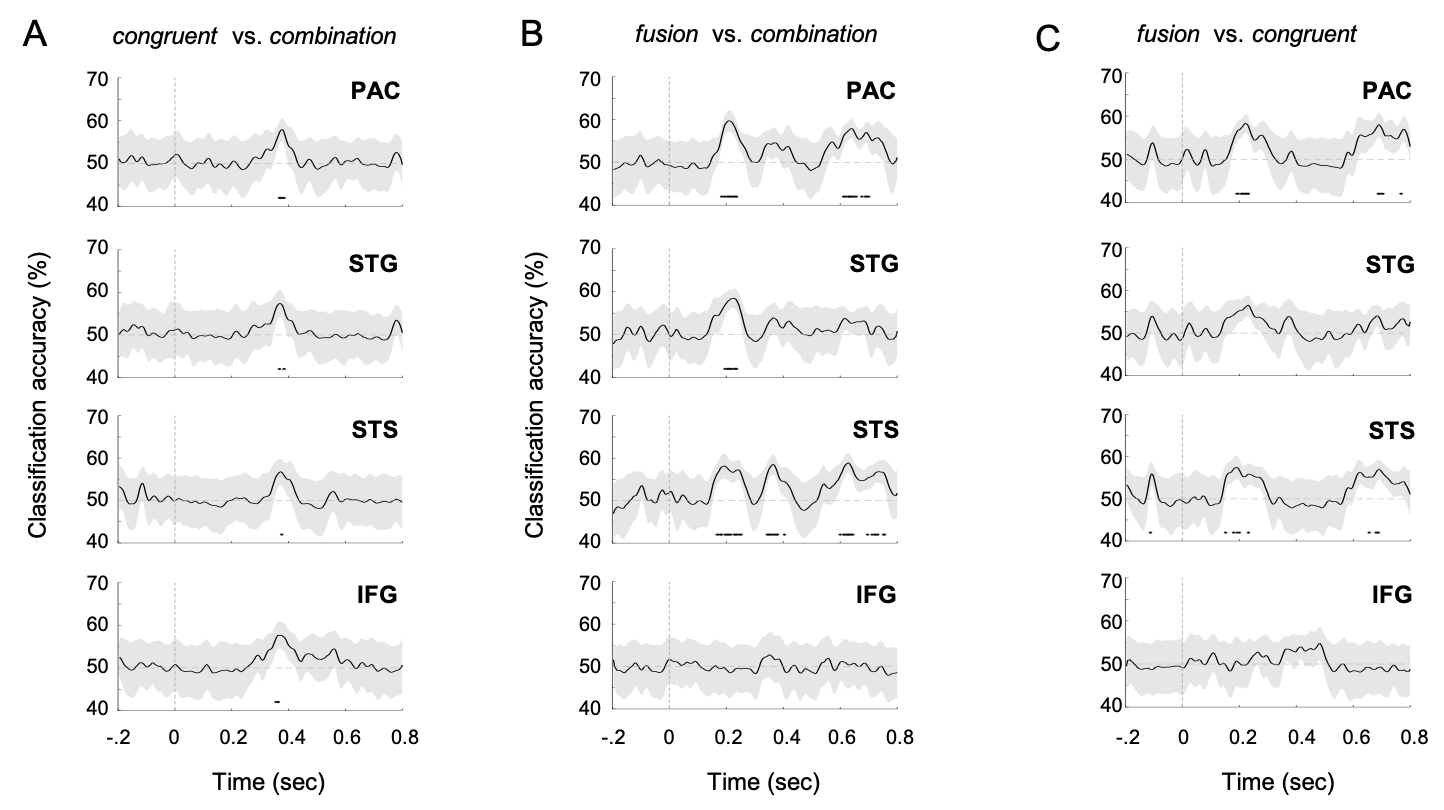


**S1 Table.** Response times (in seconds) and percentage of responses (in brackets) averaged across participants, for each category of syllables produced in experiment 1, according to each condition (congruent, fusion, and combination).

|  |  | Conditions | | |
| --- | --- | --- | --- | --- |
|  |  | Congruent | Fusion | Combination |
| Responses | aba / apa | 1.74 (3.2%) | 1.50 (30.9%) | 1.59 (6.0%) |
|  | ada / ata | 1.44 (78.8%) | 1.48 (45.5%) | 1.69 (10.2%) |
|  | aga / aka | 1.62 (10.8%) | 1.55 (12.7%) | 1.74 (21.3%) |
|  | agba / akpa | 1.79 (1.6%) | 1.76 (1.6%) | 1.62 (19.5%) |
|  | abga / apka | 1.77 (1.0%) | 1.66 (1.3%) | 1.58 (24.0%) |

**S2 Table.** Percentage of responses (mean, and standard deviation in brackets) averaged between participants, for each category of syllables, according to the speaker gender used in the video (male or female) and according to each condition (congruent, fusion, combination), in experiment 1.

|  |  | Speaker Gender | Conditions | | |
| --- | --- | --- | --- | --- | --- |
|  |  |  | Congruent | Fusion | Combination |
| Responses | aba | Female | 3.33 (0.54) | 44.33 (1.13) | 14.50 (1.13) |
|  |  | Male | 3.83 (0.38) | 16.50 (1.15) | 34.67 (1.15) |
|  | apa | Female | 2.00 (0.37) | 32.83 (1.06) | 9.83 (1.06) |
|  |  | Male | 3.50 (0.37) | 30.00 (0.98) | 26.33 (0.98) |
|  | ada | Female | 73.50 (1.09) | 39.00 (0.56) | 5.17 (0.56) |
|  |  | Male | 71.33 (1.16) | 51.33 (0.45) | 5.83 (0.45) |
|  | ata | Female | 73.50 (0.97) | 48.83 (0.65) | 6.17 (0.65) |
|  |  | Male | 68.83 (1.11) | 42.67 (0.59) | 6.83 (0.59) |
|  | aga | Female | 5.83 (0.37) | 6.67 (1.21) | 19.67 (1.21) |
|  |  | Male | 8.33 (0.55) | 15.33 (0.53) | 6.00 (0.53) |
|  | aka | Female | 13.83 (0.53) | 8.00 (1.57) | 27.67 (1.57) |
|  |  | Male | 15 (0.75) | 20.83 (0.81) | 11.50 (0.8) |
|  | agba | Female | 1.17 (0.12) | 1.67 (1.62) | 37.33 (1.62) |
|  |  | Male | 1.33 (0.14) | 2.17 (0.80) | 15.17 (0.80) |
|  | akpa | Female | 0.33 (0.05) | 0.50 (1.54) | 23.50 (1.53) |
|  |  | Male | 1.33 (0.13) | 0.83 (1.01) | 15.83 (1.01) |
|  | abga | Female | 1.67 (0.19) | 1.33 (0.86) | 12.83 (0.85) |
|  |  | Male | 2.50 (0.27) | 1.83 (1.10) | 19.67 (1.10) |
|  | apka | Female | 0.33 (0.05) | 2.17 (1.20) | 16.83 (1.20) |
|  |  | Male | 1.83 (0.23) | 1.00 (1.12) | 28.5 (1.12) |

**S3 Table.** Behavioural results for the MEG experiment. In the upper part of the table are the results of the ANOVA. In the lower part of the table are the results of the linear regression analysis between each condition and the asynchronies.

| MEG Experiment | d.f. | F | *p* – threshold with *Bonferroni* correction: 0.005 |
| --- | --- | --- | --- |
| Continuum | 1, 14 | 0.98 | 0.34 |
| Voice | 1, 14 | 0.33 | 0.57 |
| Asynchronies | 11, 154 | 0.87 | 0.57 |
| Conditions | 2, 28 | 15.99 | 0.0001* |
| Continuum*Voice | 2, 28 | 0.04 | 0.96 |
| Continuum*Asynchronies | 11, 154 | 0.63 | 0.80 |
| Continuum*Conditions | 2, 28 | 0.99 | 0.38 |
| Voice*Asynchronies | 11, 154 | 1.15 | 0.33 |
| Voice*Conditions | 2, 28 | 0.68 | 0.51 |
| Asynchronies*Conditions | 22, 308 | 1.97 | 0.0063 |
|  |  |  |  |
|  | β | t | p |
| Congruent | -0.003 | -0.519 | 0.604 |
| Combination | 0.020 | 1.815 | 0.070 |
| Fusion | -0.003 | -0.772 | 0.440 |
